# Alice im digitalen Wunderland: pädiatrische Lehre in der COVID-19-Pandemie: Eine Umfrage und Stellungnahme der AG Lehre der Deutschen Gesellschaft für Kinder- und Jugendmedizin (DGKJ)

**DOI:** 10.1007/s00112-020-01076-7

**Published:** 2020-12-02

**Authors:** Martin Häusler, Hans Martin Bosse, Thomas Fischbach, Norbert Graf, Jürgen‑Christoph von Kleist-Retzow, Joachim Kreuder

**Affiliations:** 1 Sektion Neuropädiatrie und Sozialpädiatrie, Klinik für Kinder- und Jugendmedizin, Uniklinikum RWTH Aachen, Pauwelsstr. 30, 52074 Aachen, Deutschland; 2 Klinik für Allgemeine Pädiatrie, Neonatologie und Kinderkardiologie, Universitätsklinikum Düsseldorf UKD, Moorenstr. 5, 40225 Düsseldorf, Deutschland; 3 Berufsverband der Kinder- und Jugendärzte, BVKJ e. V. Köln, Mielenforster Str. 2, 51069 Köln, Deutschland; 4 Klinik f. Päd. Onkologie und Hämatologie, Universitätsklinikum, Universität des Saarlandes, Campus Homburg, Gebäude 9, 66421 Homburg, Deutschland; 5 Klinik und Poliklinik für Kinder- und Jugendmedizin, Universitätsklinik Köln, Kerpener Str. 62, 50937 Köln, Deutschland; 6 Zentrum für Kinderheilkunde und Jugendmedizin, Universitätsklinikum Gießen und Marburg, Feulgenstr. 10–12, 35385 Gießen, Deutschland

**Keywords:** Unterricht am Patienten, Medizindidaktik, Digitale Lehre, Medizinstudium, IT-Infrastruktur, Bedside teaching, Medical didactics, E‑learning, Undergraduate teaching, IT infrastructure

## Abstract

Die Umstellung der pädiatrischen Lehre im Medizinstudium von Präsenzveranstaltungen auf digitale Formate war und ist für Lehrende und Studierende eine besondere Herausforderung. Basierend auf einer Umfrage unter pädiatrischen Universitätskliniken fasst die AG Lehre der DGKJ im folgenden Beitrag bisherige Erfahrungen zur digitalen Lehre im Sommersemester 2020 zusammen. Die Umfrageteilnehmer von 17 pädiatrischen Universkliniken berichteten über ein umfangreiches Spektrum oft kurzfristig entwickelter digitaler Lehrformate. Dabei waren Vorlesungen und Seminare gut, Unterrichtsformate mit direktem Patientenbezug und Fertigkeiten‑/Kommunikationstrainings deutlich weniger gut durch digitale Formate ersetzbar. Die Implementierung digitaler Unterrichtsformate führte zu einem deutlich erhöhten Zeitaufwand für die Lehrenden. Digitale Lehrangebote benötigen eine gute IT-Infrastruktur, sie sollten in ein Curriculum eingebunden sein und durchweg direkte Austauschmöglichkeiten zwischen Studierenden und Lehrenden vorsehen. Lehrenden sollten Schulungen in Didaktik zur digitalen Lehre und zu IT-Kenntnissen angeboten werden. Diese Ergebnisse decken sich mit der Literatur zu digitalen Lehrformaten allgemein sowie zur Lehre im Rahmen der COVID-19-Pandemie im Besonderen. Die hier vorgestellten Erfahrungen sollen nicht nur die Entwicklung digitaler pädiatrischer Lehrformate während der bestehenden Pandemie erleichtern, sondern auch darüber hinaus die Konzeption neuer digitaler Lehrangebote für die Pädiatrie im Medizinstudium anregen. Insbesondere müssen neue digitale Ersatzformate für den Unterricht am Patienten entwickelt werden.

*Die Stellungnahme wurde im Konsens von der AG Lehre der DGKJ erarbeitet und vom DGKJ-Vorstand verabschiedet.*

**Zusatzmaterial online:**

Die Online-Version dieses Beitrags (10.1007/s00112-020-01076-7) enthält Tabellen mit Freitext-Antworten der teilnehmenden Einrichtungen. Sie fassen deren Erfahrungen mit digitaler Lehre im Sommersemester 2020 zusammen und resümieren ihre Empfehlungen. Beitrag und Zusatzmaterial stehen Ihnen auf www.springermedizin.de zur Verfügung. Bitte geben Sie dort den Beitragstitel in die Suche ein, das Zusatzmaterial finden Sie beim Beitrag unter „Ergänzende Inhalte“.

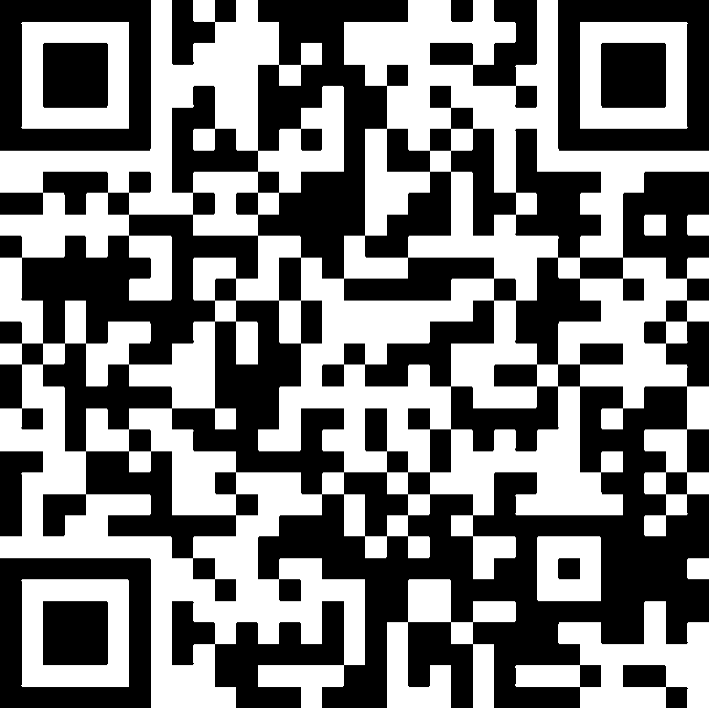

## Abstract (en)

The COVID-19 pandemic led to a rapid switch from undergraduate classroom teaching to online-teaching; a challenging process for teachers and students. Based on a recent online survey among German pediatric university hospitals the “AG Lehre der DGKJ” (teaching working group of the German Society of Pediatrics and Adolescent Medicine) summarizes latest experiences with e‑learning during the summer term of 2020. The survey participants from 17 pediatric university hospitals report that the large spectrum of e‑learning formats could sufficiently replace classical lectures and seminars but could not fully replace teaching involving direct contact to patients. The introduction of new digital teaching formats is time-consuming, needs high-quality IT infrastructure, should be embedded in a continuous curriculum and provide the possibility of regular exchange between students and teachers. Teachers should be provided with the opportunity for training in didactic methods and IT skills. These results correspond to the literature on e‑learning in general and undergraduate medical education during the COVID-19 pandemic in particular. The experiences summarized here should not only facilitate the development of e‑learning tools during the ongoing pandemic but also stimulate to establish e‑learning as a valuable component of future pediatric medical education. New digital substitutes for teaching involving pediatric patients need to be developed.

The statement was drafted by consensus by the German Society of Pediatrics and Adolescent Medicine Working Group on Teaching and approved by the DGKJ board.

## Einleitung

Die SARS-CoV-2-Pandemie stellt die universitäre Lehre vor erhebliche Herausforderungen. Es gilt, die Inhalte verschiedener Formen von Präsenzveranstaltungen in auf Distanz basierende Lehrformate umzuwandeln, mit möglichst wenig Verlust an Lehrinhalten und unter Zuhilfenahme meist nicht fakultätsweit etablierter technischer Verfahren. In der Pädiatrie tritt als weiteres Problem hinzu, dass die Veröffentlichung von Videoaufnahmen kranker Kinder besonderen Restriktionen unterliegt. Zudem ist die klinische Bewertung erkrankter Kinder aufgrund ihres ganzheitlichen Ansatzes in starkem Maße auf die direkte Interaktion mit der erkrankten Person angewiesen. Eine Rückkehr zu normalen Lehrverhältnissen ist zumindest für das Wintersemester 2020/2021 nicht möglich, und manche digitale Lehrformate sind es womöglich wert, auf Dauer in die universitäre Lehre integriert zu werden. Vor diesem Hintergrund führte die AG Lehre der DGKJ eine umfangreiche Befragung an Universitätskinderkliniken zur Lehrsituation im Sommersemester 2020 durch. Im Folgenden werden die Ergebnisse dieser Befragung zusammengefasst, mit dem Ziel, bisherige Erfahrungen zur digitalen Lehre in der Pädiatrie strukturiert zur Verfügung zu stellen, um damit die Planung der Lehre im WS 2020/2021 zu erleichtern. Zudem können diese Erfahrungen Impulse für die Entwicklung dauerhafter digitaler Lehrformate geben.

*Diese Stellungnahme wurde im Konsens von der AG Lehre der DGKJ erarbeitet und vom DGKJ-Vorstand verabschiedet.*

## Methodik

Die Arbeit basiert auf einem strukturierten webbasierten Fragebogen zu Vorlesungen, Seminaren, Blockpraktika, Unterricht am Patienten, Kommunikations- und Fertigkeitentrainings sowie zu Prüfungsformaten. Bei Vorlesungen bzw. Seminaren wurde beispielsweise differenziert nach synchronen digitalen Vorlesungen/Seminaren (*Live-Stream*), aufgezeichneten hinterlegten Vorlesungen/Seminaren (Videocast), vertonten Präsentationen (Screencast), Podcasts, hinterlegten Vorlesungen/Seminaren in Form von PDF-Dokumenten, analogem strukturiertem Selbststudium, digitalem Selbststudium (Lern-Apps, Online-Tests zum eigenen Üben), Web-Konferenzen ohne (direkt) bzw. mit vorausgehender definierter Online-Selbstlernphase als Vorbereitung sowie anderen Formaten gefragt.

Direktionen und Abteilungsleitungen universitärer Kinderkliniken und studentische Vertretungen aller medizinischen Fakultäten der Bundesrepublik wurden angeschrieben und gebeten, die Fragen im Zeitraum vom 13.08. bis 06.09.2020 mit den Lehrbeauftragten ihrer Kliniken zu beantworten. Nach Abschluss standen Antwortsammlungen von 17 Universitätskinderkliniken (Aachen, Berlin, Bochum, Bonn, Düsseldorf, Frankfurt, Freiburg, Gießen, Hamburg, Hannover, Heidelberg, Homburg, Köln, Lübeck, Marburg, Münster und Tübingen) zur Verfügung. Der Rücklauf der parallel bei Studierenden erhobenen Daten war für eine Auswertung zu gering. Der Onlinefragebogen wurde mittels SurveyMonkey (https://www.surveymonkey.de) umgesetzt. Hierbei kamen sowohl strukturierte Fragen zu den verschiedenen Lehrformaten mit Einfach- und Mehrfachantwortmöglichkeiten als auch Freitexte zum Einsatz. Die Datenerhebung erfolgte in anonymisierter Form, die Datenanalyse mittels Microsoft 365 beinhaltete lediglich eine deskriptive Auswertung.

## Ergebnisse

### Lehrformate in verschiedenen Lehrveranstaltungen

**Figure 1:**
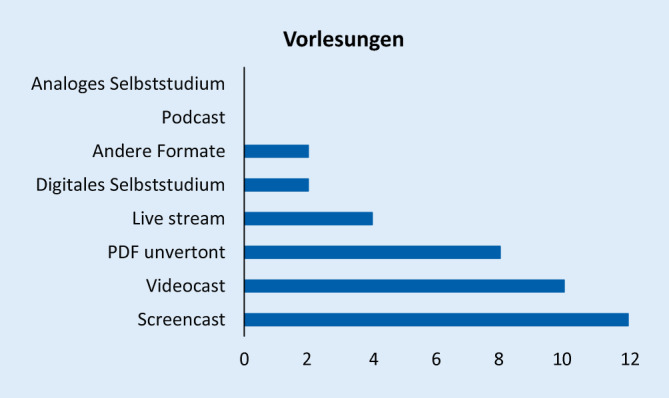

**Figure 2:**
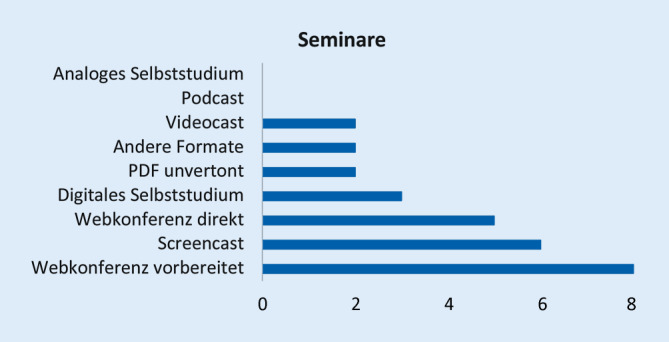

**Figure 3:**
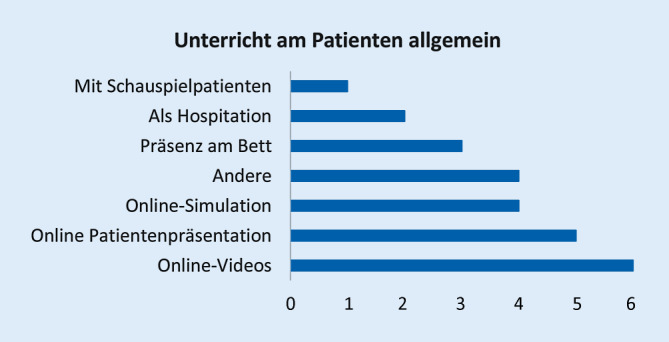

**Figure 4:**
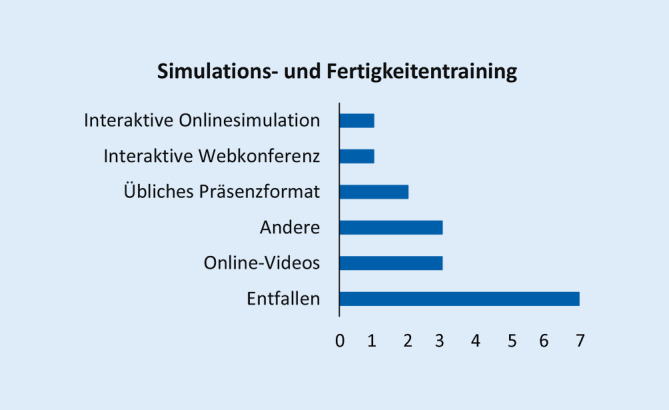

**Figure 5:**
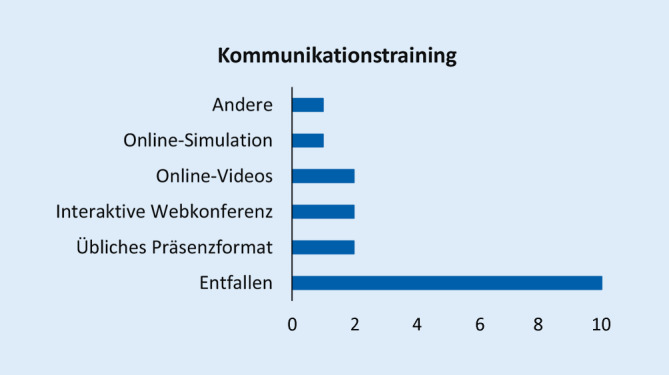

Abb. 1, 2, 3, 4, 5, 6 und 7 fassen die in verschiedenen Veranstaltungen verwendeten Lehrformate zusammen (Erläuterungen zu Abkürzungen und Fachbegriffen im Glossar). Vorlesungen, Seminare und Unterricht am Patienten wurden kaum als Präsenzveranstaltungen durchgeführt (Abb. 1, 2 und 3). Zudem erfolgten meist keine synchronen Online-Veranstaltungen, sondern es wurden Materialien wie Videos zum Selbststudium zur Verfügung gestellt. Simulationstrainings und Kommunikationstrainings entfielen häufig, auch das Blockpraktikum erfolgte kaum als Präsenzveranstaltung (Abb. 4, 5 und 6). Dies stellte insgesamt ein deutlich reduziertes pädiatrisches Lehrangebot bezogen auf klinische Fertigkeiten für die Studierenden dar. Prüfungen im Fach Kinder- und Jugendmedizin (F12) und im Blockpraktikum Kinder- und Jugendmedizin wurden weitestgehend durchgeführt, wobei für die Prüfung im Fach Kinder- und Jugendmedizin überwiegend auf das übliche Format der Präsenzklausur unter Beachtung der Hygienevorschriften zurückgegriffen wurde. Im Blockpraktikum Kinder- und Jugendmedizin kamen zahlreiche unterschiedliche Prüfungsformate zur Anwendung, wobei typische Formate, wie Präsenz-OSCE oder arbeitsplatzbasierte Prüfungen nur vereinzelt angewendet wurden (Abb. 7). Die Aussagekraft der Prüfungen im Fach Kinder- und Jugendmedizin insgesamt wurde von zwei Dritteln der antwortenden Einrichtungen als gut bis sehr gut bewertet, während bei den Prüfungen zum Blockpraktikum dies nur bei einem Drittel der Antworten gegeben war.

**Figure 6:**
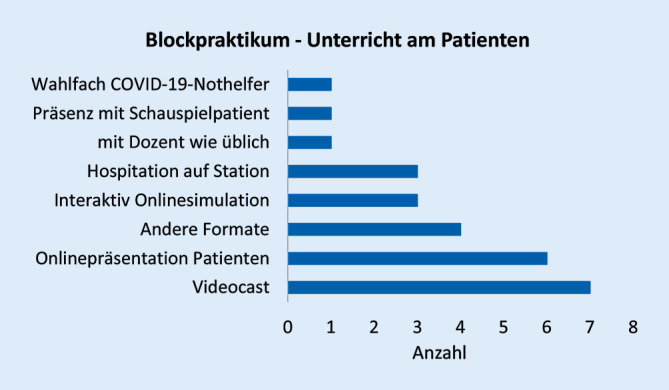

**Figure 7:**
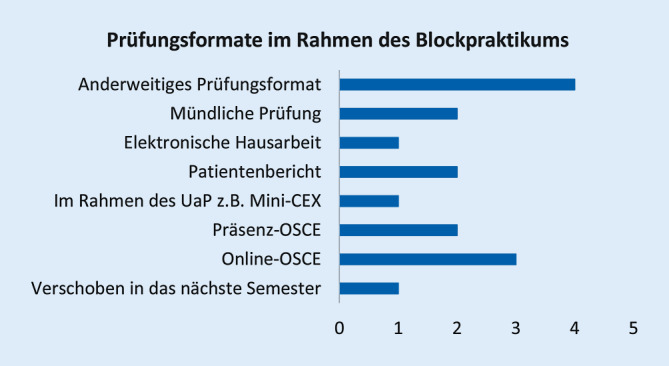

#### Infobox 1 Empfehlungen für digitale Lehrangebote

*Technik*

- Digitale Lehrangebote benötigen eine gute IT-Infrastruktur: Hardware, Software, personelle Unterstützung durch IT.

*Curriculare Einbindung*

- Digitale Lehrangebote sollten klar in ein Curriculum eingebunden sein
- und durchweg direkte Austauschmöglichkeiten zwischen Studierenden und Lehrenden vorsehen.
- Es müssen neue, geeignetere digitale Ersatzformate für den Unterricht am Patienten entwickelt werden.

*Lehrende*

- Ein deutlich erhöhter, mindestens doppelter Zeitaufwand muss für Lehrende eingeplant werden.
- Lehrenden sollten Schulungen in Didaktik zur digitalen Lehre und zu IT-Kenntnissen angeboten werden.

### Analyse der Freitexte

Tab. 1, 2, 3 und 4 fassen die Freitext-Antworten der teilnehmenden Einrichtungen zu den Fragen zusammen, was gelungen/förderlich oder nicht gelungen/schwierig war, und resümieren ihre Empfehlungen. Sie sind als ergänzendes Material online verfügbar (Zusatzmaterial online).

Bezüglich der *Vorlesungen* (Tab. 1) ergab sich kein einheitliches Votum für eine Präferenz von synchronen Online-Vorlesungen oder dem Hinterlegen von Vorlesungsvideos zum Selbststudium. Die fehlende reale Interaktion zwischen Lehrenden und Studierenden wurde nach den technischen Problemen als häufigste Negativ-Erfahrung mitgeteilt. Demzufolge wurde unabhängig vom Lehrformat eine zusätzliche Möglichkeit zur Interaktion mit den Dozierenden gewünscht (begleitende Online-Seminare, zusätzlicher Präsenzunterricht, Chat, Fragestunden). Inhaltlich wurde das Fehlen von Patientinnen‑/Patientenvorstellungen, u. a. aufgrund rechtlicher Probleme sowie die Schwierigkeit, eine Erfolgskontrolle durchzuführen, bedauert. Insgesamt bestand große Zufriedenheit mit dem Format der Online-Vorlesung als Möglichkeit der Lehre. Verbesserungsmöglichkeiten wären beispielsweise die persönliche Anwesenheit einiger weniger Studierender im Hörsaal während der Vorlesung sowie die parallele Aufzeichnung der Vorlesung als Lernmaterial. Da Online-Unterricht für viele Dozentinnen und Dozenten eine neue Erfahrung darstellt, könnten technische und didaktische Schulungen, u. a. zum Umfang und zur Strukturierung der Lehrmaterialien, von Nutzen sein.

*Online-Seminare* (Tab. 2) wurden grundsätzlich als sinnvolles Lehrformat benannt, und auch die Zufriedenheit mit Online-Seminaren seitens der Lehrenden war groß. Allerdings wurde die Teilnahme seitens der Studierenden nicht durchweg als gut bezeichnet. Als Voraussetzung für ein gelungenes Online-Seminar wurde genannt, dass sich Studierende, z. B. durch vorab verfügbares Material oder durch eine vorausgehende Vorlesung, darauf vorbereiten konnten. Die Sicherstellung einer Interaktionsmöglichkeit zwischen Studierenden und Dozierenden, z. B. durch reduzierte Gruppengrößen und Nutzung digitaler Interaktionsmöglichkeiten (Chat, Hand heben, Kommentare) wurde als wichtig erachtet. Der hohe Organisationsaufwand für dieses Lehrformat kann die Freistellung von Dozierenden und die Einstellung studentischer Hilfskräfte erfordern.

*Unterricht am Patienten* (Tab. 3), d. h. Präsenzunterricht an realen erkrankten Kindern und Jugendlichen, konnte nach durchgängiger Einschätzung nicht zufriedenstellend durch ein Online-Format (oder Schauspielpatientinnen und -patienten) ersetzt, sondern allenfalls unterstützt werden. Als Behelfsformate wurden Unterricht am Krankenblatt, Fallvignetten, webbasierte Seminare oder „Best-practice“-Kurzvideos verwendet. Hilfreich könnten hierzu institutsübergreifende Datenbanken sein, für deren Einrichtung jedoch unter anderem datenschutzrechtliche Fragen zu klären sind. Auch die Rückkehr zum Unterricht am Patienten unter noch zu definierenden krankenhaushygienischen Voraussetzungen (vorherige SARS-CoV-2-Testung der Studierenden) wurde angeregt.

Bezüglich des *Simulations- und Fertigkeitentrainings* (Tab. 4a) wurde durchgängig angeführt, dass begleitende Videos und aktuelle digitale Veranstaltungen grundsätzlich hilfreich sind. Auch können sie dazu beitragen, Lehrkonzepte zu vereinheitlichen. Den Präsenzunterricht können sie jedoch nicht ersetzen. Die Ausstattung mit Lehrmaterialien für den Simulationsunterricht wurde häufiger als unzureichend beschrieben. Erfolgversprechend schien am ehesten ein integriertes Konzept aus angeleiteter Selbstlernphase mit Video-Material, synchronem Online-Unterricht und Präsenz-Training in kleinen Gruppen zu sein.

Bezüglich des *Kommunikationstrainings* (Tab. 4b) wurde wenig Erfahrung mitgeteilt, vermutlich bedingt durch den häufigen Ausfall dieses Unterrichts. In der Tendenz schien es schwierig, dieses durch Online-Formate zu ersetzen. Positive Erfahrung wurde mit einer Video-Konferenz mit Patienten gesammelt. Retrospektiv wurde festgestellt, dass bei ausreichender Planungsmöglichkeit häufiger auch Präsenzunterricht in Kleingruppen hätte stattfinden können.

Bezüglich des *Blockpraktikums* wurde zweimal geantwortet, dieses unter Berücksichtigung der Hygieneempfehlungen erfolgreich in Präsenz durchgeführt zu haben. Weitere 6 gaben an, es mit positiven Erfahrungen durch Online-Formate ersetzt zu haben. Als problematisch wurden für die Online-Veranstaltungen fehlender direkter Kontakt zu den Studierenden und das Fehlen praktischer Übungen genannt. Als Lösung wurde vorgeschlagen, zusätzlich zu Online-Angeboten wieder Präsenzlehre anzubieten, bei Online-Seminaren kleine Gruppengrößen zu wählen und die Dozentinnen und Dozenten zu schulen.

*Technische Voraussetzungen*. Durchgängig wurde als wichtige und häufig noch zu verbessernde allgemeine Voraussetzung für die verschiedenen Online-Formate eine funktionierende Technik (Hardware, Software, personelle Unterstützung durch IT) benannt.

## Schlussfolgerung und Empfehlungen

Digitale Formate werden bereits seit vielen Jahren für das Medizinstudium entwickelt, wobei dies jedoch häufig für spezielle Fragestellungen erfolgte, wie die Verschreibung von Medikamenten, das Erlernen pädiatrischer Reanimationstechniken, die Bearbeitung umweltmedizinischer Fragen oder spezielle dermatologische oder physiologische Lehrinhalte [1–6]. Mit dem Auftreten der SARS-CoV-2-Pandemie wurde es jedoch erforderlich, Online-Unterricht nicht nur für einzelne, sondern für alle und sehr verschiedene Lehrveranstaltungen anzubieten, wobei Lehre, die traditionell der Interaktion mit Patientinnen oder Patienten bedarf, eine besondere Herausforderung an Lehrende und Studierende darstellt. Pädiatrische Lehre am Patienten beinhaltet eine zusätzliche Problemstellung, da die Veröffentlichung von Videos mit Kindern nur unter besonders strengen datenschutzrechtlichen Bestimmungen möglich ist.

Bereits vor der COVID-19-Pandemie befasste sich eine Vielzahl von Untersuchungen mit Vor- und Nachteilen sowie den Voraussetzungen für gelungene digitale Lehrformate. Als Vorteile wurden beispielsweise folgende Faktoren identifiziert: eine Erweiterung des Spektrums an Lehrtools für die Lehrenden sowie die Möglichkeit, Lehrinhalte rasch an sich ändernde Bedürfnisse anpassen zu können; die Stimulation aktiven Lernens bei den Lernenden unter anderem aufgrund der Modernität des digitalen Formates; die Möglichkeit, unabhängig von Ort und Zeit zu lehren; die Möglichkeit, Wissen mittels Videos aus dem Alltag sehr praxisnah vermitteln zu können; die Möglichkeit, digitale Medien zu Vorbereitung, Unterstützung und Nachbereitung des Präsenzunterrichts einzusetzen. Dazu muss der Lehrinhalt auf die sonstigen Lernziele abgestimmt sein und sollte die Studierenden inhaltlich und zeitlich (10–15 min maximal) nicht überfordern. Als wichtig zeigte sich zudem, dass sich die Studierenden motiviert, mit Selbstdisziplin und mit Flexibilität auf das digitale Lehrmedium einlassen können, dass das Lehrmaterial pädagogisch gelungen ist, die Beteiligten über technische Vorkenntnisse verfügen und adäquate technische Voraussetzungen vorliegen. Weitere Faktoren, die die Zufriedenheit der Studierenden mit digitalen Lehrformaten beeinflussen, sind die Leistungsansprüche der Studierenden an sich selbst sowie das Lernklima. Gleichzeitig sind digitale Lehrformate nach bisherigen wissenschaftlichen Analysen zeit-, kosten- und arbeitsintensiv und weniger geeignet für die Vermittlung praktischer Fähigkeiten [7–9]. Bei der Erstellung der Videos sollte darauf geachtet werden, für welchen Zweck und für welchen Ausbildungsstand der Studierenden sie gedacht sind, und darauf, ob sie als alleiniges Lehrmaterial oder zur Ergänzung, z. B. zur Vorbereitung auf nachfolgende Präsenzlehre, fungieren. Sinnvoll ist es, Interaktionsmöglichkeiten innerhalb der Anwendungen (z. B. Quiz) oder mit den Dozierenden während oder nach dem Angebot des elektronischen Lehrmediums vorzusehen [1, 5, 6, 9].

Die im Rahmen dieser Umfrage erhobenen Daten spiegeln dieses Wissen in hohem Umfang wider. Dabei waren insbesondere Vorlesungen und Seminare gut durch Online-Formate ersetzbar. Auch die hier beschriebenen Erfahrungen unterstreichen erneut die große Bedeutung von Interaktionsmöglichkeiten zwischen Studierenden und Dozierenden, sowohl vor (Bereitstellung von Materialien zur Vorbereitung in Lernräumen), während als auch nach der synchronen Online-Veranstaltung, beispielsweise durch Chats oder zusätzliche Seminare. Lehrmaterialien sollten nicht ohne eine Austauschmöglichkeit mit den Dozentinnen und Dozenten zum ausschließlichen Selbststudium hinterlegt werden. Zudem sollten, um die Interaktionsmöglichkeiten in Seminaren zu verbessern, die Gruppengrößen begrenzt werden. Mit diesen Maßnahmen sollte es möglich sein, eine deutlich höhere Zahl an Live-Online-Veranstaltungen durchzuführen, als dies in den teilnehmenden Fakultäten im SS 2020 erfolgte (siehe Empfehlungen in den Tabellen).

Für das Simulationstraining, den klassischen Unterricht am Patienten, für das Kommunikationstraining und das Blockpraktikum wurden keine zufriedenstellenden virtuellen bzw. digitalen Ersatzmöglichkeiten beschrieben. Synchrone oder asynchrone Online-Vorlesungen und Lehrvideos konnten die Erfahrung aus der direkten Interaktion mit Patientinnen und Patienten nur unzureichend ersetzen. Insbesondere für das Blockpraktikum liegen jedoch nun begrenzte Erfahrungen vor, die zeigen, dass der Unterricht am Patienten hier unter Einhaltung der Hygieneregeln durchaus häufiger als Präsenzveranstaltung durchgeführt werden könnte.

Häufig wurde für die hier beschriebene neue Lehrsituation ein deutlich erhöhter, mindestens doppelter Zeitaufwand für die Dozierenden festgestellt, der letztlich eine vermehrte Freistellung der Leistungsträger für die Lehre erfordert. Dieser Zeitaufwand liegt beispielsweise in der Erstellung neuer Lehrmaterialien begründet. Sollte zunehmend Kleingruppenunterricht oder Einzellehre am Patienten erfolgen, würde der Lehraufwand zusätzlich deutlich ansteigen.

Unverzichtbar ist für alle digitalen Lehrformate eine fundierte technische Unterstützung, durch Software, Hardware und professionelle personelle Hilfestellung.

In aktueller, zur COVID-19-Pandemie publizierter Fachliteratur fanden sich verschiedene Kommentare zur aktuellen Lehre an Hochschulen, die die im Rahmen der hier vorgestellten Umfrage gemachten Erfahrungen ebenfalls bestätigen. Dies betrifft die Bedeutung funktionierender Technik (Breitband-Internet, Software, Hardware) [10, 11]; die Vertrautheit mit der Technik seitens der Studierenden und der Lehrenden [9]; die Vermeidung inhaltlich und zeitlich zu umfangreicher Lehreinheiten [9, 10]; die Einbeziehung der Studierenden über Entscheidungs- und Diskussionsmöglichkeiten in die Lehrmaterialien bzw. Lehrveranstaltungen [12–14]; die Verfügbarkeit von Materialien zur Vorbereitung auf eine spätere Online-Veranstaltung [9, 12, 14]; direkte Feedback-Möglichkeiten während einer Online-Lehrveranstaltung [13, 15, 16]; möglichst kleine Gruppen bei Seminaren [13]; die Möglichkeit, sich auch nach einer Veranstaltung weiter mit den Dozierenden oder den Studierenden thematisch auszutauschen (z. B. Chat) [12, 16]; den hohen personellen und organisatorischen Aufwand zur Etablierung von Kleingruppenseminaren [13] sowie die Berücksichtigung rechtlicher Aspekte [9]. Als wichtig wurde in der Literatur ebenfalls das Vorhandensein eines strukturierten Curriculums benannt, um Redundanzen zu vermeiden, um den Schwierigkeitsgrad an den Kenntnisstand der Studierenden anzupassen und um die Präsentation nicht relevanter Lehrinhalte zu vermeiden [13]. Unterricht am Patienten sollte so intensiv wie möglich unter Beachtung der notwendigen Schutzmaßnahmen erhalten und genutzt werden [17, 18]; die kollegiale Zusammenarbeit mit Akademischen Lehrkrankenhäusern, mit kinderärztlichen Primärversorgern (Praxen) sowie zwischen den Disziplinen innerhalb einer Fakultät und zwischen Fakultäten sollte gestärkt werden [19].

Weitere, zum Teil detaillierte aktuelle Empfehlungen sind in folgenden Arbeiten zusammengefasst: [9–12, 14, 16, 20–25]. Über die „International Association for Medical Education“ (AMEE) wurden im Rahmen der COVID-19-Pandemie ebenfalls Ressourcen und weiterführende Empfehlungen zum digitalen Unterricht zusammengestellt (https://amee.org/covid-19).

Unter rechtlichen Aspekten sind zwei Probleme für die pädiatrische Lehre von besonderer Bedeutung: die Einbindung von Videos und Bildern pädiatrischer Patientinnen und Patienten vor dem Hintergrund der DSGVO und die Einbindung nicht selbst erstellter Inhalte in eigene Lehrmaterialien. Für die erste Problematik müssen nach aktuellem Kenntnisstand noch detaillierte Regularien entwickelt werden. Bezüglich der zweiten Problematik kann auf das Gesetz zur Angleichung des Urheberrechts an die aktuellen Erfordernisse der Wissensgesellschaft – Urheberrechts-Wissensgesellschafts-Gesetz (UrhWissG) vom 1. September 2017 verwiesen werden (Neuregelungen der Urheberrechtsgesetze (UrhG) im Bereich der Bildung und Wissenschaft in den §§ 60a bis 60h). Demnach dürfen für den Unterricht bzw. die Lehre an Universitäten (Präsenzlehre und E‑Learning) bis zu 15 % eines veröffentlichten Werkes vervielfältigt, verbreitet oder öffentlich zugänglich gemacht werden, solange kein kommerzielles Interesse besteht. Voraussetzungen für eine Einbindung einzelner Abbildungen und Textauszüge in eigene Vorlesungsmaterialien sind eine inhaltliche Auseinandersetzung mit dem zitierten Material und eine korrekte Urheber- und Quellenangabe (Angaben ohne Gewähr).

Die hier vorgestellten Ergebnisse bilden keine abgeschlossene Empfehlung, sondern fassen erste Erfahrungen als allgemeine Hilfestellung zusammen. Diese unterliegen den vor Ort verfügbaren Möglichkeiten und müssen sich an den Verlauf der Pandemie anpassen. Wichtig erscheint die Feststellung, dass eine Umstellung der Lehre vom Präsenzbetrieb in einen weitgehend digitalen Unterricht in der Pädiatrie im Sommersemester 2020 aufgrund des sehr hohen Engagements aller Beteiligten rasch und zufriedenstellend umgesetzt werden konnte. Dies darf jedoch nicht darüber hinwegtäuschen, dass die akut bereitgestellten digitalen Lehrmöglichkeiten einer langfristigen Unterstützung auf der Ebene digitaler Infrastrukturen und der Personalentwicklung bedürfen und insbesondere für den Unterricht am Patienten noch adäquate Ersatzformate zu entwickeln sind.

## Caption Electronic Supplementary Material





## Glossar

Asynchrone Lehre: Die Studierenden lernen anhand eines vorab durch die Lehrenden fertiggestellten Lehrmediums.
*Blended learning*: Kombination aus Präsenzveranstaltungen und digitaler Lehre
DGSVO: Datenschutzgrundverordnung
Digitale Lehre: Lehrformate basierend auf digitalen Medien
Live-Stream: Eine Lehrveranstaltung wird synchron online zu den Studierenden übertragen.
Mini-CEX: Mini Clinical Evaluation Exercise
OSCE: Objective Structured Clinical Examination
Podcast: Reines Audioformat einer Lehrveranstaltung zum Selbststudium hinterlegt.
Präsenzveranstaltung: Studierende befinden sich persönlich bei der Patientin/dem Patienten oder im Hörsaal/Unterrichtsraum.
Screencast: Vertonte Präsentationen wurden zum Selbststudium hinterlegt.
Synchrone Lehre: Die Studierenden bekommen die Lehrinhalte zeitgleich von den Lehrenden vermittelt.
UaP: Unterricht an dem Patienten/der Patientin
Videocast: Vertonte Videos wurden zum Selbststudium hinterlegt.
