# Supplementary material for: Alice in the digital wonderland—pediatric teaching during the COVID-19 pandemic: A survey and comment of the Teaching Working Group of the German Society of Pediatrics and Adolescent Medicine (DGKJ)
Source: Monatsschr Kinderheilkd. 2020 Dec 2;169(2):151–8. [Article in German] doi: 10.1007/s00112-020-01076-7 (PMC7709092; doi:10.1007/s00112-020-01076-7)
Supplement: Supplementary file 1 [file 112_2020_1076_MOESM1_ESM.pdf]

Tabelle 1: Freitextkommentare zu Vorlesungen

Thema	Was war gelungen/förderlich (n=20)	Was war nicht gelungen/schwierig (n=22)	Empfehlungen (n=18)
Technik per se	Technikausstattung gut (3/3)	Probleme mit Hard- und / oder Software (7/7)<br>Fehlender Live-Stream (1/1)	Technische Ausstattung bzw. Support verbessern (3/3)
Technische / Zentrale Unterstützung	Guter technischer Support (6/6)	Fehlende technischer Support (5/5)	Technischen Support allgemein (2/2) bzw. bei Erstellung Videos verbessern (1/1)
Organisation	Videos zum Selbststudium: Größerer Nutzerkreis als bei Online-Vorlesungen (2/2)	Probleme Copyright (1/1)<br>Probleme Datenschutz (1/1)<br>Probleme Studierende zu Veranstaltungen einzuladen (1/1)	Lernplan, um Kumulation am Semesterende zu vermeiden (1/1)<br>Soviel Präsenzvorlesung wie möglich einrichten (1/1).
Dozentinnen/Dozenten	Hohes Engagement einiger Dozierender (1/1)<br>Dozentinnen/ Dozenten zufrieden (1/1)	Dozentinnen/Dozenten mit neuen Formaten sehr gefordert (1/1)<br>Dozentinnen/Dozenten unzufrieden (1/1)	Schulungen didaktisch / technisch für Dozentinnen/Dozenten (3/3) und Studierende (1/1).<br>Hohen Vorbereitungsaufwand bedenken (1/1)
Gestaltung Unterricht	Online-Vorlesungen, (2/2)<br>Vorlesungsvideos für Selbststudium, (4/4)<br>Online-Vorlesungen für zusätzliches Selbststudium aufgenommen<br>Inhalte auf wesentliches komprimiert (1/1)	Fehlen von Patientinnen/Patienten (2/2)<br>Vorlesungsvideos schlechter als Online-Vorlesungen (1/1) bzw. umgekehrt (1/1)<br>Hochgeladene Inhalte zu umfangreich (1/1), zu unstrukturiert (1/1) oder zu spät verfügbar (1/1)	Online-Vorlesungen sinnvoll (8/8). Verbesserbar durch Präsenz einiger Studierender (1/1), parallele Aufzeichnung für Selbststudium (2/2), sowie didaktische Weiterentwicklung (1/1). Videocasts verbessern durch begleitende Online-Seminare (2/2), Präsenzunterricht (1/1) und Diskussionsforen (1/1)
Zufriedenheit Studierende	Zufriedenheit gut (9/9)		
Interaktion Dozierende / Studierende	Dozentin/Dozent via Chat (2/2) oder Fragestunde (1/1) für Rückfragen erreichbar	Fehlende Interaktion<br>Studierende mit Dozentinnen/Dozenten (5/5)<br>Dozierende untereinander (1/1)	Kontakt zu Studierenden allgemein erhalten bzw. verbessern (2/2)
Lernerfolg	Lernerfolg gut (4/4)	Keine Kontrolle von Lernerfolg möglich (1/1)<br>Lernerfolg schlecht (1/1)	
Teilnahme	Gut (1/1)	Fehlende Kontrolle (1/1)	

Tabelle 2: Freitextkommentare zu Seminaren

Thema	Was war gelungen/förderlich (n=20)	Was war nicht gelungen/schwierig (n=18)	Empfehlungen (n=14)
Technik	Einfach technisch umsetzbar (2/6)<br>Stabiles Internet, Datenrate (1/2)<br>Zentrale Unterstützung (4/6)	Technische Ausstattung/Umsetzung (4/6)<br>Internet, Datenrate (1/2)<br>Zentrale Unterstützung (2/6)<br>Hoher Schulungsaufwand (Digitale Lehrerfahrung sinnvoll) (3/3)	Verbesserung der Technik und Support (3/3)<br>Beherrschung der Technik und Software, um alle Funktionalitäten nutzen zu können (1/1)
Organisation		Akkumulation am Semesterende (1/1)<br>Hoher Organisationsaufwand (2/2)<br>Zu hohe Belastung der Lehrkoordinatoren (1/1)	Hilfe durch studentische Tutoren (1/1)
Dozentinnen/Dozenten	Großes Engagement (1/2)	Fehlende didaktische Fertigkeiten (1/2)	Freistellung für Lehre (1/1)
Gestaltung des Unterrichts	Interaktiver Unterricht (3/6)<br>Diskussionsmöglichkeit (4/4)<br>Multimediale Materialien, Lehrvideos, Videocasts (2/2)<br>Interdisziplinär (1/1)<br>Fallvignetten (2/2)<br>Webinare (1/1)	Fehlende Interaktion (3/6)<br>Fehlende Selbstlernphase (3/3)<br>Praktischer Unterricht sehr erschwert (1/1)	Begleitende Webinare (2/2)<br>Videocasts (1/1)<br>Mehr Interaktion (1/1)<br>Regelmäßige Online-Fragestunden (1/1)<br>Kombination Selbstlernphase und Online-Unterricht (2/2)
Gruppengröße	Kleine Gruppen (1/1)	Große Gruppengröße (1/1)	Kleinere Gruppengröße (2/2)
Zufriedenheit	Generelle Zufriedenheit (5/5)<br>Hohe Akzeptanz (2/2)<br>Positive Evaluation (1/1)	Fehlendes Vorwissen (2/2)<br>Geringe Teilnahmequote (1/1)	
Prüfung		Freitextaufgaben zu zeitaufwendig (1/1)	
Lernerfolg	Lernerfolg bei der Bearbeitung von Fällen hoch (3/3)		
Teilnahme		Kontrolle der Teilnahme sehr schwierig (1/1)	Videos der Studierenden sollen eingeschaltet sein (1/1)

Tabelle 3: Freitextkommentare zu Unterricht am Patienten

Thema	Was war gelungen / förderlich (n=16)	Was war nicht gelungen / schwierig (n=19)	Empfehlungen (n=14)
Technik	Gute Technik (1/4)	Technische Probleme (3/4), fehlendes Equipment	Ausbau Technik (2)
Organisation		Vorbereitungszeit und hoher Aufwand (2/2); Aufteilung zwischen Disziplinen, Datenschutz bei Online-Unterricht am Patienten	Datenschutzfragen klären; Institutsübergreifende Datenbanken und Materialsammlungen schaffen; Finanzielle Ressourcen erhöhen; Didaktikschulungen
Gestaltung des Unterrichts	Jede Form einer doch gelingenden Präsenz-Lehre (4/4), zT mit vorheriger SarsCOV2-Testung<br>Neue Formate: interaktive Fallvignetten, Beschreibung einer körperlichen Untersuchung durch Studierende, Unterricht am Krankenblatt, Kurzvideos<br>Best practice: Kurzvideos „Corona TV – Pädiatrie in 90 Sekunden"<br>Live-online-Begleitung durch Webinare	UaP in Präsenz im Grunde nicht ersetzbar (8); (weitere 2 gleiche Kommentare bei Frage 3.1)<br>Schauspielpatientinnen und -patienten erreichen nicht Lernerfolg und Lernzufriedenheit von echten Patientinnen/Patienten	konventioneller UaP in Präsenz nicht ersetzbar (6)<br>Ggfs. vorherige SarsCOV2-Testung Studierende
Zufriedenheit	Zufriedenheit gut in 5/5	Studentisches Feedback fehlt	
Teilnahme		Keine rege Nutzung von Online-Materialien	

Tabelle 4a: Freitextkommentare zu Simulations-/Fertigkeitentraining

Thema	Was war gelungen / förderlich (n=7)	Was war nicht gelungen / schwierig (n=7)	Empfehlungen (n=9)
Technik/Ausstattung mit Lehrmaterialien	Zentrale Unterstützung (1/2)	Unzureichende Ausstattung mit Lehrmaterialien (1/2)	Kann grundsätzlich nicht digital durchgeführt werden (1/1)
Organisation		Zu knappe Planungszeit (1)<br>Ausgefallen (2)	
Gestaltung des Unterrichts	Neue Videos erstellt (1/2)<br>einheitliches Lehrkonzept entwickelt (1/1)<br><br>Einsatz von Videos, um Interaktion in der Kleingruppe zu fördern (1/1)	Hoher Aufwand in Vorbereitung (1/2)<br>Unterricht an Simulationspuppen kann nicht digital abgebildet werden (1/1)<br>Fehlende direkte Lernkontrolle bei Üben der Fertigkeit (1/1)	Erstellen von mehr Videos sinnvoll (2)<br>Interaktive Online-Videos mit Fragen sind unterstützend sinnvoll (1)<br>Untersuchung von Kleinkind und Säugling nur in Real-Präsenz vermittelbar (1)<br>Zentrale Datenbank der DGKJ wünschenswert (1)<br>Begleitende web-Seminare (2)<br>Videocasts (2)<br>Kombination Selbstlernphase und Online-Unterricht + Präsenz-Training in kleinen Gruppen (1)
Zufriedenheit	Generelle Zufriedenheit (3/4)	Generelle Unzufriedenheit (1/4)	Keine positive Empfehlung möglich (1)
Lernerfolg/Lehrqualität	Verbesserter Lernerfolg u.a. durch konsistentes Konzept (1/2)<br>Lernerfolg hoch (1/5)	Lernerfolg stark abhängig von Motivation der Kleingruppe (1/2)<br>Lernerfolg durch fehlenden Präsenz-Unterricht sehr ungenügend (4/5)	Kann in Bezug auf den angestrebten Lernerfolg nicht/kaum digital durchgeführt werden, muss in Präsenz erfolgen, Präsenz nicht ersetzbar (4)

Tabelle 4b: Freitextkommentare zu Kommunikationstraining

Thema	Was war gelungen / förderlich (n=7)	Was war nicht gelungen / schwierig (n=7)	Empfehlungen (n=7)
Technik/Ausstattung mit Lehrmaterialien		Technische Umsetzung sehr schwierig, deshalb darauf verzichtet (1/1)	
Organisation	konkreter Ablaufplan sehr gut trotz kurzfristiger Information durch Fakultät (1/2)	Sehr kurzfristige Info zum Ablauf (1/2)<br><br>Ausgefallen (2)	Präsenz-Unterricht bei ausreichend großem Raum und kleiner Gruppengröße doch möglich (1)<br>Frühzeitig planen (1)
Dozentinnen/ Dozenten	Hohe Bereitschaft zum Engagement (1/2)	Engagement eingeschränkt durch zu wenig Zeit und unzureichende Ressourcen (1/2)	
Gestaltung des Unterrichts	Zum Thema „Anamnese" Nutzung von Fallvignetten als Ersatz für Videos (1/2)<br><br>Simulation einer Videokonferenz/-Sprechstunde mit Patientinnen und Patienten (1/1)	Verzicht auf Videos mit Schauspielpatientinnen und -patienten, da zu hoher Zeitaufwand und Feedbackrunde/Selbstreflexion nicht umsetzbar (1/2)<br>Nur sehr kleiner Teil des üblichen Kommunikationstraining möglich (1/1)	Mehr Videos geplant, insbesondere zu Fallpräsentation, Patientenübergabe, Kommunikation mit Eltern (1)<br>Kommunikation im Präsenzbetrieb notwendig (1)
Gruppengröße			Max. 8 Stud. pro Gruppe (1)
Zufriedenheit	Zufriedenheit groß (2/2)		Keine positive Empfehlung möglich (1)
Lernerfolg/ Lehr-Qualität	Erstaunlich hoher Lernerfolg (1/2)<br>Video-Konferenz mit Patientinnen/Patienten sehr gut (1/1)	Geringer Lernerfolg (1/2)<br>Geringere Authentizität (1/1)	Muss in Präsenz erfolgen/nicht ersetzbar (1)
Teilnahme der Studierenden		Sehr gering, da keine Pflichtveranstaltung (1/1)	Pflichtteilnahme notwendig wegen schlechter Akzeptanz (auch vor Corona) (1)
